# Antigen-Sparing and Enhanced Efficacy of Multivalent Vaccines Adjuvanted with Immunopotentiators in Chickens

**DOI:** 10.3389/fmicb.2017.00927

**Published:** 2017-05-26

**Authors:** Peipei Wu, Jihu Lu, Lei Feng, Hongzhuan Wu, Xuehua Zhang, Mei Mei, Jibo Hou, Xiufan Liu, Yinghua Tang

**Affiliations:** ^1^National Research Center of Engineering and Technology for Veterinary Biologicals, Jiangsu Academy of Agricultural SciencesNanjing, China; ^2^Key Laboratory of Veterinary Biological Engineering and Technology, Ministry of Agriculture, Jiangsu Academy of Agricultural SciencesNanjing, China; ^3^Jiangsu Co-Innovation Center for the Prevention and Control of Important Animal Infectious Disease and ZoonosisYangzhou, China; ^4^Department of Biological Sciences, Alabama State University, MontgomeryAL, United States; ^5^Animal Infectious Disease Laboratory, College of Veterinary Medicine, Yangzhou UniversityYangzhou, China

**Keywords:** adjuvant, immunopotentiator, monovalent vaccine, polyvalent vaccine, vaccine sparing, antigen sparing, sterile immunity

## Abstract

We previously described that immunopotentiators, CVCVA5, increased the efficacy of H5 and H9 subtype avian influenza vaccines in chickens, ducks, and geese. In this study, we further investigated the effects of the CVCVA5 for improving the efficacy of other univalent or multivalent inactivated vaccines. The immune response administrated with half-dose of monovalent vaccine plus CVCVA5 were higher than those of one dose of monovalent vaccine without immunopotentiators as measured by levels of antibodies from serum, tears and bronchoalveolar lavage fluids, and cytokines of IFNγ and IL-4 from serum. Vaccines included the univalent vaccine of Newcastle Disease virus (ND), Egg Drop Syndrome virus (EDS), Infectious Bronchitis virus (IB), and Infectious Bursal Disease virus (IBD). The CVCVA5 also improved the immune response of both ND and IBD vaccines with less dosage. The sterile protective immunity was monitored with one- or a half-dose of adjuvanted ND vaccine or one dose of adjuvanted IBD vaccine, respectively. The improved immune efficacy was observed in a half-dose of adjuvanted bivalent vaccines compared to one dose of vaccines without CVCVA5 as measured by the antibody levels, including bivalent vaccine of ND-H9, ND-IB, and ND-IBD. The CVCVA5 also boosted the immune efficacy of the tetravalent vaccine (ND-IB-EDS-H9). A half-dose of adjuvanted commercial vaccine or 75% antigen-sparing adjuvanted vaccine elicited similar antibody levels to those of one dose non-adjuvanted commercial vaccines. The CVCVA5 improved the effect of a booster vaccination as measured by the antibody levels against H5 or H9 virus antigens, in which chickens primed with the adjuvanted ND-IB vaccines given a booster with H5–H9 bivalent vaccines without CVCVA5 using 5-day intervals. The inflammatory response may contribute to these additional effects by increasing the levels of IFNγ and IL-4 after the injection of the adjuvanted ND-IB vaccines. Results indicated that the CVCVA5 improved the serum and mucosal antibody levels, cytokine levels of the chickens given the univalent vaccine, and also improved serum antibody titers in bivalent and tetravalent vaccines. This has a potential as an improve vaccine.

## Introduction

Contagious viral diseases are a constant threat to the poultry industry. Vaccines are an effective strategy to prevent the spread of diseases. Newcastle disease virus (NDV) and avian influenza virus (AIV) (Tang et al., 2009; Alexander et al., 2012; Kapczynski et al., 2013), and infectious bronchitis virus (IBV) may cause diseases at any age (Chhabra et al., 2015). In contrast infectious bursal disease virus (IBDV) is more common in 3–15 weeks old chickens (Mahgoub et al., 2012). Therefore, vaccination programs must be customized according to the diseases found on the poultry farms.

Combined multivalent inactivated viral emulsion vaccines are widely employed in poultry farms because of savings in time and labor, and reduced stress caused by manual handling during immunization (Fukanoki et al., 2001; Lemiere et al., 2011). Multivalent vaccines included the bivalent vaccine, ND vaccine combined with H9 subtype AI (H9) vaccine (ND-H9), ND vaccine with IB vaccine (ND-IB), ND vaccine with IBD vaccine, and the tetravalent vaccine, combination of ND, IB, H9 vaccine and egg drop syndrome (EDS) vaccine (ND-IB-EDS-H9).

To keep the same antigen contents similar to those in monovalent vaccines for each single component in multivalent vaccine, the antigen stock solution should be concentrated during the manufacturing processes of the multivalent vaccines. The volume of single dose vaccine also limits the number of the antigen components in multivalent vaccine. Therefore, it is important to have balance between maintaining the potency and maximum numbers of the antigen in multivalent vaccines.

Inactivated multivalent vaccines have a disadvantage similar to the monovalent inactivated vaccine that lack danger signals molecules, such as dsRNA, ssRNA, and CpG. These signal molecules can elicit a more robust immune response via activation of innate immune pathway (Coffman et al., 2010). Various strategies have been adopted to increase the immunological efficacy of the inactivated viral emulsified vaccine, such as the addition of the immunopotentiators (Fox et al., 2013). The adjuvant, CVCVA5, showed the effects on improving efficacy of both the serum and mucosal antibody response and the cell mediated immune response of inactivated vaccine in chickens (Tang et al., 2014; Lu et al., 2016). The components of CVCVA5 contain ligands to pattern recognition receptors, poly I:C, resiquimod, imiquimod, muramyldipeptide (MDP), and chemicals with immune enhancement activity, levamisole hydrochloride. The poly I:C is the ligand of toll-like-receptors (TLRs)-3 and RIG-I-like receptors (RLR). While the resiquimod, imiquimod are the ligands of TLR-7/8 (Vasilakos and Tomai, 2013). The MDP is recognized by NOD-like receptors (NOD)-2 (Sonoda et al., 2000). Levamisole is an antiparasitic agent and is also capable of immune enhancement (Oladele et al., 2012).

There are no reports which studied the immunopotentiators which improving the efficacy of multivalent animal vaccines. We compared the efficacy of H5 and (or) H9 vaccines with or without CVCVA5 in both specific pathogen-free (SPF) and commercial chickens (Tang et al., 2014; Lu et al., 2016). In this study, we systematically studied the efficacy of CVCVA5 on various inactivated vaccines. The use of the CVCVA5 in chickens has increased the immune response of both univalent and polyvalent vaccines, generated sterile immunity with the monovalent vaccine of ND and IBD, assisted in reducing vaccine of both univalent and polyvalent vaccines, and facilitated antigen sparing of tetravalent vaccine in field application.

## Materials and Methods

### Cell and Viruses

The DF-1 cell (ATCC No: CRL-12203) derived from American Type Culture Collection were cultured in Dulbecco’s Modified Eagle Medium containing 10% fetal calf serum (Gibco). H9 subtype AIV strain, A/Chicken/SD/YH01/2011 (SDYH01), was isolated from chickens (Tang et al., 2014). IBDV strain of SD01, used in serum neutralization (SN) test (Vakharia et al., 1994), was isolated and characterized. The common virus strains used for evaluating the vaccine efficacy were derived from Chinese Institute of Veterinary Drug Control (Beijing), including standard strains, La Sota (NDV), and Chinese virulent strain, F48E8 (NDV), M41 strain (IBV), very virulent strain BC6/85 (CVCC AV7, IBDV) and AV127 strain (EDSV). These viruses were propagated in 10- to 11-day old specific pathogen-free (SPF) chicken embryonated eggs, and the EDS viruses were inoculated in 10- to 11-day-old SPF duck embryos. The allantoic fluids of infected embryos were collected, aliquoted and stored at -80°C. The viral antigen of M41 strain used for hemagglutinin inhibition (HI) assay was derived from GD Animal Health, Netherlands. At end of experiments, the discarded live viruses and infected animal carcasses were autoclaved and incinerated to eliminate biohazards.

### Vaccines and Adjuvants

If not mentioned, the following inactivated monovalent and multivalent vaccines were commercial products, including the monovalent EDS vaccine (Jing 911 strain, Tianbang Bio-industry Co. LTD, Nanjing), IBD vaccine (D78 strain, MSD Animal Health, Shanghai), ND vaccine (La Sota strain, Tianbang), bivalent vaccine ND-H9 (La Sota strain + HP strain, Ringpu Biological Co. LTD, Tianjing), ND-IB (Clone 30 strain + M41 strain, MSD) and ND-IBD (La Sota strain + HQ strain, Yikang Biological Co. LTD, Liaoyang), and trivalent vaccine ND-IB-EDS-H9 (La Sota strain + M41 strain + AV127 strain + NJ02 strain, Tianbang).

The inactivated IB monovalent vaccine (M41 strain) was prepared according to the criteria of multivalent vaccine, which contains the component of M41 strain, 10^6.5^ mean egg infective dose (EID_50_) per dose, and the live IB commercial vaccine (H120 strain) was derived from Tianbang.

The CVCVA5 was prepared as oil emulsion adjuvants as reported in a previous report (Tang et al., 2014). Briefly, the CVCVA5 was prepared as water-in-oil emulsions which is similar to the form of the inactivated vaccine. The CVCVA5 composed of L–D isoform MDP (InvivoGen), poly I:C (InvivoGen), levamisole hydrochloride (Sigma) in aqueous phase and resiquimod (InvivoGen) and imiquimod (InvivoGen) in oil phase. The oil emulsion form of CVCVA5 was mixed with the inactivated vaccine (v:v, 1:9) before vaccination.

### CVCVA5 Combination Used with the Monovalent Vaccine in SPF Chickens

The adjuvant efficacy of CVCVA5 in the univalent vaccines, ND, IBD, or EDS, was determined on groups of 3-day-old SPF chickens (*n* = 20). Chickens were immunized subcutaneously with one dose (0.3 ml) or half-dose (0.15 ml) of the above-mentioned vaccines with or without CVCVA5. One group of chickens was set as control.

Seven groups of SPF chickens (*n* = 20) were used to test the efficacy of IB vaccine with or without CVCVA5. Chickens in the first group were primed with a single dose (containing 10^3.5^ EID_50_ in 0.1 ml) of live attenuated IB vaccine (H120) via intranasal administration in 2-day old chickens, and subcutaneously boosted with one dose (0.3 ml) of inactivated IB vaccine (M41) at 16 days of age. The second group received the similar prime-boost vaccination regime as those chickens in the first group except for modification of the M41 vaccine mixing with CVCVA5. Chickens in the third and fourth groups were subcutaneously inoculated with a single dose of M41 vaccine with or without CVCVA5 at 16 days of age, respectively. The fifth group was administered a half-dose of M41 vaccine with CVCVA5. The sixth group was vaccinated intranasally with a single dose of live H120 vaccine, and the seventh group was control.

Blood samples from all birds and tears samples (Orr-Burks et al., 2014) from three birds of each group were collected at 2, 3, and 4 weeks post-last vaccination weeks (PV). The bronchoalveolar lavage fluids (BAL) from three birds of each group were also collected at 4 weeks PV (Lu et al., 2016). Chickens in groups of ND vaccine which received with either no adjuvants or adjuvants were challenged intramuscularly (IM) with 10^5.0^ mean egg lethal dose (ELD_50_) of F48E8 virulent virus, respectively. The chickens which received IBD vaccine with or without adjuvants were challenged with 10 mean bursal infective doses (BID_50_) of BC6/85 virus at 4 weeks PV via the intraocular route, respectively. In the ND virus challenge groups, the clinical signs of all birds were monitored for 14 days. Oropharyngeal and cloacal swab samples were collected at 3, 5, and 7 days post-challenge (PC) for virus isolation. While in the IBD virus challenge groups, the monitoring period was only 3 days, and the swab samples were collected at 3 days PC.

### Effects of the CVCVA5 on the Minimal Dosage of Monovalent ND and IBD Vaccine in SPF Chickens

Groups of 3-day-old SPF chickens (*n* = 15) were used to test the efficacy of CVCVA5 on the minimal dosage of the univalent ND and IBD vaccine. One dose (0.3 ml), 1/4th (0.075 ml), 1/8th (0.038 ml),1/16th (0.019 ml), or 1/32nd (0.010 ml) dose of ND or IBD vaccine mixed with the water-in-oil emulsion form of CVCVA5 (0.1 ml), or mixed with the similar water (PBS only)-in-oil emulsion (0.1 ml) as control, respectively. Chickens were bled at 2, 3, and 4 weeks PV.

### CVCVA5 Combination Use with the Bivalent Vaccines in the Commercial Chickens

The role of CVCVA5 in commercial bivalent vaccine, ND-H9, ND-IB, and ND-IBD, were assessed in 10- to 14-day-old commercial chicken (Hy-Line Brown). Groups of 15 chickens were subcutaneously immunized with a single dose (0.3 ml) or half-dose (0.15 ml) of those bivalent vaccines with or without CVCVA5. Serum samples were collected at 2, 3, and 4 weeks PV.

### CVCVA5 Mixing with the Antigen Sparing Tetravalent Vaccines in Field Application

The antigen contents in one standard dose of commercial tetravalent vaccine (ND-IB-EDS-H9) included 10^8.5^ EID_50_ of ND viruses, 10^6.3^ EID_50_ of IB viruses, 10^3^ hemagglutinin units of EDS viruses and 10^7.5^ EID_50_ of H9 viruses. The antigen-sparing tetravalent vaccine contained only one quarter of antigen content in one dose of vaccine as compared to those in the standard commercial vaccine. The commercial or antigen-sparing tetravalent vaccine given with or without CVCVA5 were evaluated in the same poultry farm as separate commercial flocks (200 to 300 birds per flock), 110- to 120-day-old layers via IM route.

The chickens in the first and second flocks were injected with one dose (0.5 ml) of standard commercial tetravalent vaccine with or without CVCVA5, respectively. Birds in the third and fourth flocks were given a half dose (0.25 ml) of commercial tetravalent vaccine with or without CVCVA5, respectively. The antigen-saving tetravalent vaccine with or without CVCVA5 was administered to the fifth and sixth flocks, respectively. A random selection of birds was bled (*n* = 10 to 15) at 2, 3, and 4 weeks PV.

### Effects of CVCVA5 Adjuvant on an Administrated Vaccine

We also investigated the effects of the CVCVA5 on the immune response of the subsequently injected inactivated vaccines with 5-day interval in the field. Two flocks (800 to 900 birds per flock) of Hy-Line brown breed chickens were administrated ND-IB vaccines with or without CVCVA5 at 5 days of age, and then both flocks received a commercial bivalent H5–H9 vaccine at 10-day-old. The 5-day interval between immunization of ND-IB and H5–H9 vaccines is based on the immune program of poultry farm. Fifteen chickens from each flock were randomly selected for serum collection (*n* = 10 to 12) to test antibody at 2, 3, and 4 weeks PV. At 5 and 14 days PV with ND-IB vaccines, the cytokine levels of IFNγ and IL-4 were determined from pooled serum samples of each flock.

### Titration of Serum and Mucosal Antibody Titer and Cytokine Levels

Serum antibody levels were titrated by HI assay for EDSV, IBV, and NDV, or by SN test for IBDV with DF-1 cell line (Tang et al., 2013). The cytokine levels of IFNγ (Invitrogen, CA) and IL-4 (USCN, Wuhan, China) in chickens serum of pooled samples were detected by commercially available ELISA kit following the manufacturer’s instructions and with reference to our previous report (Lu et al., 2016).

The IgA antibodies in pooled tear samples were monitored by ELISA. Briefly, the purified ND, EDS, IB (M41), or IBD (SD01) viruses were coated as detection antigen (5 μg/ml), respectively, and pooled tear samples were diluted fourfold. The HRP conjugated goat anti-chicken IgA (Thermo) was used as second detection antibody with 1000-fold dilutions. Furthermore, mucosal antibody levels of pooled BAL samples were monitored by HI test with three repeats for ND, IB, and EDS viruses antigen accordingly, and by SN test for IBD in DF-1 cell which similar to the test of serum antibody.

### Ethics Statement

All studies were carried out in strict accordance with the recommendations in the National Guide for the Care and Use of Laboratory Animals. The protocol (VMRI-AP140516) was approved by the Review Board of National Research Center of Engineering and Technology for Veterinary Biologicals, Jiangsu Academy of Agricultural Sciences.

### Statistics

Results were presented as means ± the standard errors (SEM). Prism software (GraphPad Software, Inc., San Diego, CA, United States) was used for data analysis with unpaired two-tailed Student *t*-test or a one-way analysis of variance (ANOVA). Comparisons used to generate *P*-values are indicated by horizontal lines (^∗^*P* < 0.05, ^∗∗^*P* < 0.01, ^∗∗∗^*P* < 0.001).

## Results

### The Adjuvants Improve the HI Antibody Levels of the Monovalent Vaccine

**FIGURE 1:**
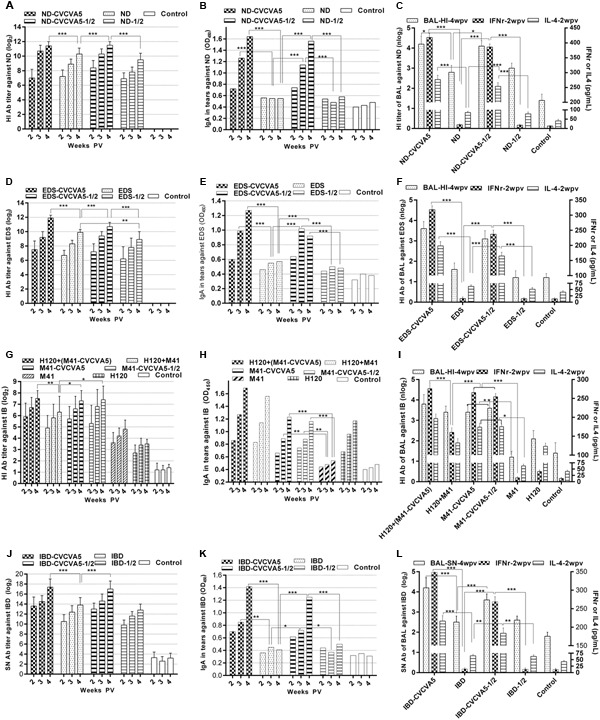
**Antibody titers against virus antigen of monovalent vaccines.** Groups of 3 days old age of SPF chickens (*n* = 20) were vaccinated with the following vaccines. ND-CVCVA5, one dose of CVCVA5 adjuvanted ND vaccine. ND, one dose of ND vaccine without CVCVA5. ND-CVCVA5-1/2, half-dose of CVCVA5 adjuvanted ND vaccine. ND-1/2, half dose of ND vaccine **(A)**. The name of EDS **(D)** and IBD **(J)** vaccines groups were analog to those of ND vaccines. H120+(M41-CVCVA5) and H120+M41, the chickens primly vaccinated one dose of live H120, and boosted with one dose of M41 with or without CVCVA5 with 7 days intervals **(G)**. Mucosal antibody levels in tears of pooled samples for ND **(B)**, EDS **(E)**, IB **(H)**, and IBD **(K)**. The HI antibody titer in BAL (*n* = 3) at 4 weeks PV (weeks post vaccination), and IFNγ and IL-4 levels in serum (*n* = 5) at 2 weeks PV for ND **(C)**, EDS **(F)**, IB **(I)**, and IBD **(L)**.^∗^*P* < 0.05, ^∗∗^*P* < 0.01, ^∗∗∗^*P* < 0.001.

Groups of chickens were administered either one dose or half-dose of ND vaccine with or without CVCVA5. Serum antibodies against ND significantly enhanced in chickens given the ND vaccine mixed with CVCVA5 when compared to those chickens, which received the ND vaccine without CVCVA5 at 2, 3, and 4 weeks PV (**Figure 1A**), respectively. Furthermore, the chickens given ND-CVCVA5 vaccine, the HI titers in the group vaccinated with half-dose (0.15 ml) were similar to the group, which received one dose (0.3 ml). However, the dose-effects were observed in the chickens injected with ND vaccine without CVCVA5. In groups of chickens, which received the commercial vaccine without CVCVA5, the HI titers of the chickens given half-dose vaccine were significantly lower than those chickens, which received with one full dose.

Enhanced efficacy was examined in the CVCVA5-adjuvanted univalent EDS or IBD vaccine in the chickens. The antibody titers in the groups, which received a half-dose of EDS or IBD adjuvanted vaccines produced similar to the levels as those chickens with one single dose of the adjuvanted vaccines, respectively, while significantly higher than those groups, which received one or half-dose of EDS (**Figure 1D**) or IBD (**Figure 1J**) vaccine without immunopotentiators.

Chickens from three groups vaccinated with CVCVA5-adjuvanted inactivated M41 vaccine had higher antibody levels than the other three groups that received no CVCVA5. This included groups with the H120 prime and M41 booster vaccine, only with a single dose of live H120 vaccine, or with a single dose of inactivated M41 vaccine only (**Figure 1G**). Chickens that received the half-dose of adjuvanted M41 vaccine group elicited similar HI antibody levels as birds given one full dose of adjuvanted M41 vaccine.

### The Adjuvants Significantly Elevated Levels of Mucosal Antibody and Cytokine of the Monovalent Vaccine

In the groups that received the univalent vaccine of ND, EDS, and IBD, the IgA levels in tears in groups at 2, 3, and 4 weeks PV (**Figures 1B,E,K**), and HI or SN antibodies in BAL at 4 weeks PV (**Figures 1C,F,L**), chickens in the adjuvanted vaccine groups were significantly higher than those that were not given adjuvants. Birds which received half dose of adjuvanted vaccine elicited significantly higher mucosal antibody than those which received full dose of vaccine without adjuvants.

Similar to the results of the ND univalent vaccine tests, the adjuvants also induced higher levels of mucosal antibody in tears (**Figure 1H**) and BAL (**Figure 1I**) with M41 IB vaccine. Chickens vaccinated with only H120 live vaccine also elicited higher mucosal antibody levels when compared to those which received the adjuvanted vaccine. However, no synergistic efficacy was detected in the chickens administered the combination of the H120 live vaccine with adjuvanted M41 vaccine in mucosal antibody level.

Results of the IFNγ and IL-4 levels in serum at 2 weeks PV were similar to those of the mucosal antibody levels. Higher levels of IFNγ and IL-4 in serum of the chickens which received the adjuvanted vaccine were detected when compared with those injected vaccine alone without immunopotentiators of the ND, EDS, IB, and IBD vaccine, respectively.

### The Adjuvants Help in Reducing the Minimal Dosage of the Monovalent ND and IBD Vaccine

**FIGURE 2:**
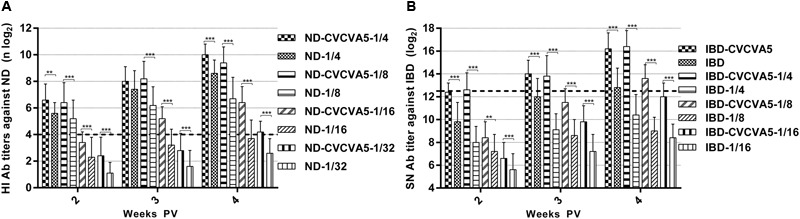
**Antibody titers in the minimal dosage of ND and IBD vaccine test.** Groups of 3-day-old SPF chickens (*n* = 15) were used to determine the efficacy of CVCVA5 on the minimal dosage of ND and IBD test. 1/4th (0.075 ml), 1/8th (0.038 ml),1/16th (0.019 ml), or 1/32nd (0.01 ml) dose of ND mixed with 0.1 ml volume of oil emulsion form of CVCVA5 or oil emulsion with only PBS in the aqueous phase as control, respectively **(A)**. One dose (0.3 ml), 1/4th (0.075 ml), 1/8th (0.038 ml), or 1/16th (0.019 ml) dose of IBD vaccine were mixed with 0.1 ml volume of the oil emulsion form of CVCVA5 or with the similar oil emulsion containing only PBS in aqueous phase as control, respectively **(B)**. Chickens were bled at 2, 3, and 4 weeks PV. ^∗∗^*P* < 0.01, ^∗∗∗^*P* < 0.001.

Determining the minimal dosage of commercial vaccine at 4 weeks PV to qualify the eligibility criteria (ND, HI ≥ 4 log2, IBD, SN ≥ 12.4 log2), 1/32nd dose was adequate for ND vaccine with adjuvants, while ND vaccine without adjuvants required 1/8th dose volume, and the 1/16 dose volume was closed to qualified efficacy (**Figure 2A**), the adjuvanted IBD vaccine needed 1/8th dose, and IBD vaccine without adjuvants needed at least 1/4th dose (**Figure 2B**).

### The Adjuvants Help to Induce Sterile Immunity for ND and IBD Vaccines

No virus was isolated from either one dose or half-dose of the adjuvanted ND vaccines group from swabs isolation from both the cloaca and the oropharynx. However, 3 out of 15 birds were positive for virus isolation which received one dose and half-dose of ND vaccine groups without adjuvants. All birds were not infected or died in the control group (**Table 1**).

**Table 1:** The Newcastle Disease virus (ND) and Infectious Bursal Disease virus (IBD) shedding post-challenge.

Challenge virus	Groups	Positive swabs of cloaca			Positive swabs of oropharynx			Total positive	Morbidity	Mortality
		3 dpc	5 dpc	7 dpc	3 dpc	5 dpc	7 dpc			
NDV	ND-CVCVA5	0/15	0/15	0/15	0/15	0/15	0/15	0/15	0/15	0/15
	ND-CVCVA5-1/2	0/15	0/15	0/15	0/15	0/15	0/15	0/15	0/15	0/15
	ND	2/15	1/15	0/15	3/15	2/15	0/15	3/15	0/15	0/15
	ND-1/2	1/15	2/15	1/15	3/15	3/15	1/15	3/15	0/15	0/15
	Control	15/15	15/15	15/15	15/15	15/15	15/15	15/15	15/15	13/15
IBDV	IBD-CVCVA5	0/15	-	-	0/15	-	-	0/15	0/15	0/15
	IBD-CVCVA5-1/2	1/15	-	-	0/15	-	-	1/15	0/15	0/15
	IBD	3/15	-	-	1/15	-	-	3/15	0/15	0/15
	IBD-1/2	5/15	-	-	1/15	-	-	5/15	0/15	0/15
	Control	15/15	-	-	15/15	-	-	15/15	15/15	0/15

*Chickens in the ND challenge groups were subcutaneously inoculated 10^*5.0*^ ELD_*50*_ of F48E8 virulent viruses at 4 weeks post vaccination (wpv). The clinical signs of all birds were monitored for 14 days. Oropharyngeal and cloacal swab samples were collected at 3, 5, and 7 days post-challenge (dpc) for virus isolation. Birds in the IBD challenge groups were inoculated intraocularly with 10 BID_*50*_ BC6/85 viruses at 4 wpv. The monitoring period was only 3 days and the swab samples were collected at 3 dpc. The morbidity was counted with the clinical signs of challenge chickens. -, not test.*

In the IBDV challenge groups, no bird had virus shedding in chickens received one dose of IBD-CVCVA5 vaccine, but one bird showed virus shedding in the half-dose of IBD-CVCVA5 vaccine. Four out of 15 chickens were positive for viral shedding that received one full dose of commercial vaccine group, and one-third (5/15) of chickens had viruses shedding from that received the half-dose of commercial vaccine group. All challenged control birds developed infection, but survived post exposure (**Table 1**).

### The Adjuvants Boosted the Antibody Levels in Birds That Received One Dose or Half-Dose of Bivalent Vaccine

Serum antibody levels were significantly enhanced in birds vaccinated with the three bivalent vaccines which combined with CVCVA5, respectively.

**FIGURE 3:**
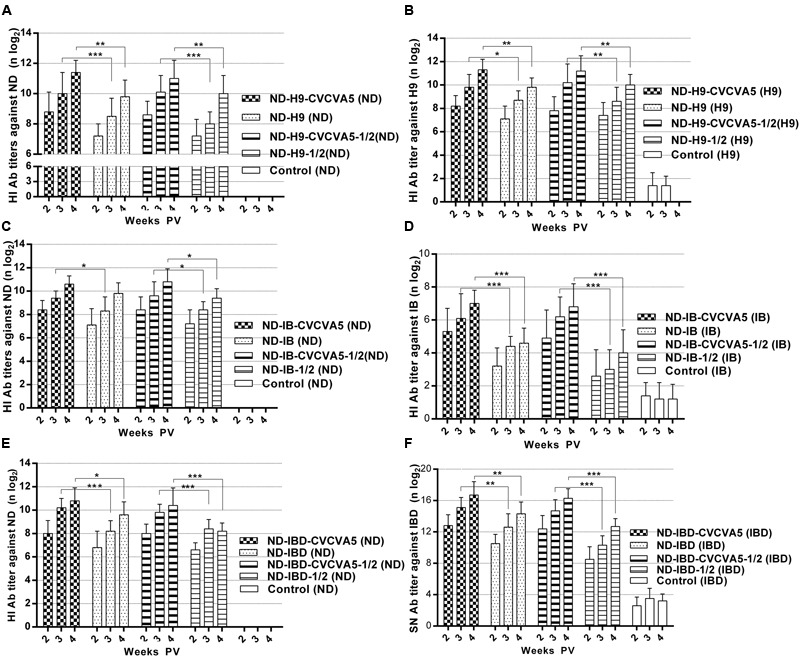
**Antibody titers against virus antigen of the bivalent vaccines.** ND-H9-CVCVA5, one dose of CVCVA5 adjuvanted ND-H9 vaccine. ND-H9, one dose of commercial ND-H9 vaccine without CVCVA5. ND-H9- CVCVA5-1/2, half-dose of CVCVA5 adjuvanted ND-H9 vaccine. ND-H9-1/2, half dose of ND-H9 commercial vaccine **(A,B)**. The name of ND-IB **(C,D)** and ND-IBD **(E,F)** vaccines groups were similar to those of ND-H9 vaccines. ^∗^*P* < 0.05, ^∗∗^*P* < 0.01, ^∗∗∗^*P* < 0.001.

In light of the HI antibody titers against ND or H9 AI antigens, the humoral immune response in groups that received adjuvants were significantly higher than groups that received no adjuvants. The HI antibody titers against ND antigens in the chickens that received one full dose or half-dose of ND-H9-CVCVA5 vaccines were increased at least 1 log2 when compared with those chickens given the corresponding one dose or half-dose of ND-H9 vaccines at 2, 3, and 4 weeks PV (**Figure 3A**). However, the antibody levels against H9 antigens were elevated 0.9 log2 (**Figure 3B**).

As expected, the adjuvants also enhanced immunological responses at both the ND-IB and ND-IBD bivalent vaccines in terms of the antibody levels. In groups that received one dose or half-dose of ND-IB-CVCVA5 vaccines at 2 weeks PV, the antibody titers against IB antigen were higher than those groups that received one dose of ND-IB vaccines without adjuvants at 4 weeks PV (**Figure 3D**). The serological immune responses of the chickens to IBDV were similar to those of the birds to IBV. The SN antibody levels against IBDV in the adjuvanted group were at least 2 log2 higher than those of the non-adjuvanted group (**Figure 3F**). Antibody titers of the chickens against NDV in both the adjuvanted ND-IB and ND-IBD bivalent vaccine groups were similar to those of birds to the adjuvanted ND-H9 bivalent vaccine groups. They were elevated approximately 1 log2 compared to those bivalent vaccines without adjuvants (**Figures 3C,E**).

### The Adjuvants Increase the Immune Response to the Antigen Sparing Tetravalent Vaccines

Tetravalent vaccines are frequently administered in some Chinese native chicken breeds which received long-term feeding period (>70 days) or replacement layers. To test the adjuvants effects on the application of the vaccine sparing or antigen sparing, groups of chickens were given half-dose of commercial vaccines with or without CVCVA5, or administered the adjuvanted or non-adjuvanted antigen-saving tetravalent vaccines, which contained only one quarter of viral antigen than those in the standard commercial vaccine.

**FIGURE 4:**
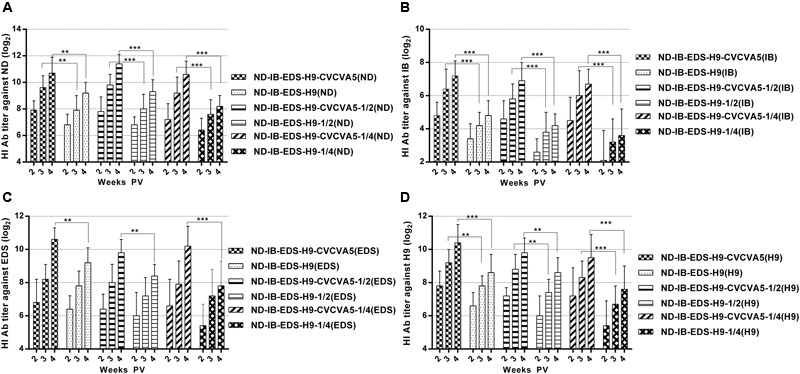
**Antibody titers elicited by the tetravalent vaccines.** ND-IB-EDS-H9-CVCVA5, one dose of CVCVA5 adjuvanted ND-IB-EDS-H9 vaccine. ND-IB-EDS-H9, one dose of commercial ND-H9 vaccine without CVCVA5. ND-IB-EDS-H9-CVCVA5-1/2, half dose of CVCVA5 adjuvanted ND-IB-EDS-H9 vaccine. ND-IB-EDS-H9-1/4, antigen-sparing vaccine containing one quarter of antigen contents as those of the ND-IB-EDS-H9 standard commercial vaccine. ND-IB-EDS-H9-CVCVA5-1/4, adjuvanted antigen-sparing vaccine. The testing antigens were ND **(A)**, IB **(B)**, EDS **(C)**, H9 **(D)**, respectively. ^∗∗^*P* < 0.01, ^∗∗∗^*P* < 0.001.

The improved effects of the CVCVA5 on the tetravalent vaccines of commercial chickens in the field were similar to those chickens, which received the univalent or bivalent vaccines, even on the antigen-sparing vaccines. According to the antibody levels against ND (**Figure 4A**), IB (**Figure 4B**), EDS (**Figure 4C**), and H9 (**Figure 4D**) antigens, respectively, the effects of CVCVA5 didn’t decrease the groups that received reducing half-dose of vaccine or three fourths of viral antigen contents compared with those groups injection with one full dose of vaccine. Serum antibody levels in groups of one dose, half-dose or 3/4 antigen-saving vaccine with adjuvants were higher than those that received one dose vaccine without adjuvants. However, without addition of adjuvants, the dose-saving or antigen-sparing tetravalent vaccines had decreased serological immune response based on the antibodies titers, especially the antibody levels against IB and EDS antigens.

### Vaccine with Adjuvants up Regulated the Antibody Response of the Subsequent Immunization

**FIGURE 5:**
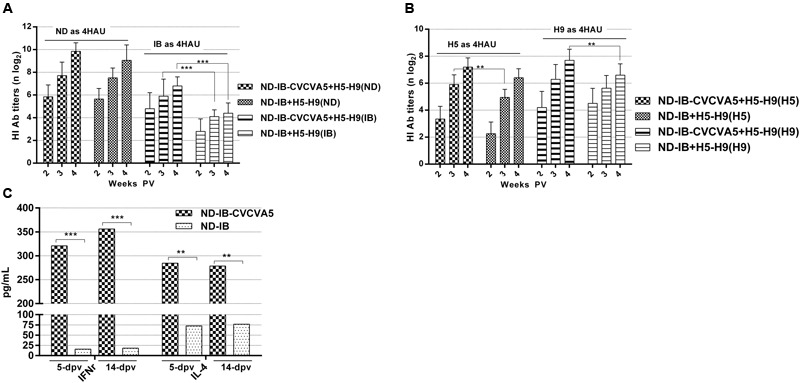
**Antibody titers against virus antigen and cytokine levels in program of immunization.** The HI titers of serum antibodies at 2-, 3- and 4-week post vaccination of H5-H9 vaccine were measured with four units of hemmaglutinin antigens of ND and IB **(A)**, H5 and H9 **(B)**. The cytokine levels of IFNγ and IL-4 in serum were tested at 5- and 14-day post vaccination of ND-IB with or without CVCVA5 **(C)**. ^∗∗^*P* < 0.01, ^∗∗∗^*P* < 0.001.

The study was carried out to test the effect of CVCVA5 adjuvant, which was given vaccines at early stage, on the subsequently immunized vaccines in the same vaccination program. The ND-IB bivalent vaccines with adjuvants were administered in chickens at day 5, and the H5–H9 bivalent vaccines that received no adjuvants administered at day 10. The antibody levels against both H5 and H9 were slightly enhanced in the flocks previously given ND-IB-CVCVA5 and boosted by the injection of H5–H9 vaccines when compared to those initially administered with ND-IB vaccine without CVCVA5 and then given the H5–H9 vaccines (**Figure 5B**). As expected, the serological immune responses to the NDV or IBV antigen were elevated in the flocks, which received with ND-IB-CVCVA5 in contrast to those birds vaccinated with only ND-IB vaccines without adjuvants (**Figure 5A**).

Both cytokine levels of the IFNγ and IL-4 were greatly improved by CVCVA5 as compared with the results from groups of ND-IB and ND-IB-CVCVA5 at 5 and 14 days PV, respectively (**Figure 5C**).

## Discussion

To save labor, time and cost, and reducing the stress due to handling birds, some owners of poultry farms mix different univalent or bivalent commercial vaccines, and simultaneously vaccinated them with partial dose or single dose of vaccine at same or different sites. However, these vaccination procedures are not uniformly efficacious, as occasionally occur in so-called “vaccine failures” as a result of administration of partial doses of vaccines, or poor adsorption of vaccines by host, because of simultaneous inoculations (Matheis et al., 2001). The available approaches to overcoming these shortcomings are to develop addition types of polyvalent vaccines or to minimize the volume of vaccine during manufacture process (Tola et al., 1999). Combination of the immunopotentiators with polyvalent vaccine is one available way to solve this issue.

Adjuvanted vaccines reduced to half-dose volume and didn’t have an influence on the efficacies of the monovalent vaccines of ND, EDS, IBD, and IB. Specifically, fewer dosages were demanded for adjuvanted ND and IBD vaccine to qualify the requirements in comparison with the non-adjuvanted vaccines. However, the efficacy of commercial vaccines without adjuvants showed the dose-effect relationship, especially EDS, IBD, and IB vaccines, in which the reduction of the dose volume caused the decrease of antibody levels and the increase of virus shedding (Iqbal, 2012).

The components of the CVCVA5 are capable of boosting innate and adaptive immune response. The poly I:C, the agonist of TLR3, is a potent inducer of innate immunity (Tatematsu et al., 2014), induce robust Th1 biased immunity, and also is capable of enhancing Th2 cytokine IL-4 (Kato et al., 2007; Jin et al., 2010). The imiquimod and resiquimod recognized by TLR7/8 are effective in activation of dendritic cells and B cells to elicit cytokines optimal for Th1 cell immunity, and antibody production. The MDP, ligand of NOD2, induce mucosal antibody response (Shafique et al., 2013), and skew a Th1-cell response and promote secretion of IFNγ. Levamisole exert effects on immune response through the activation of dendritic cells or T cell (Chen et al., 2008). Either Th1 or Th2 cells or a combination of these cell types can support mucosal immunity (Neurath et al., 2002). The effects of these agonists were further evidenced in this study.

In the efficacy study, we have detected that the monovalent vaccine combination use with CVCVA5, up regulation of IFNγ and IL-4 levels in serum, and mucosal antibodies in tears and BAL, which are also consistent with our previous study with H5 or H9 vaccine mixing use with CVCVA5 (Tang et al., 2014; Lu et al., 2016). Rising cytokine levels and mucosal immunity may provide some clues to the CVCCVA5 improving the efficacy of vaccine, including vaccine- or antigen-sparing tests, and the sterile immunity test with ND and IBD vaccination.

Bivalent vaccines are widely employed in different breeds of poultry flocks. The adjuvanted bivalent (ND-H9, ND-IB, and ND-IBD) vaccines showed similarly improved efficacy in terms of the antibody titers compared to those that received non-adjuvanted commercial vaccines. The CVCVA5 may improve the innate immune responses, which contribute to enhance immunity of the adjuvanted bivalent vaccines.

It is known that the tetravalent vaccines are the highest number of antigen containing vaccine for chickens in China. These polyvalent vaccines are widely adopted as one of the strategies to prevent and control the spread of infectious disease in intensive-farming of commercial poultry flocks. However, the manufacturing processes of polyvalent vaccines are more complicated than those of the monovalent vaccines, which involved from the steps of the filtration and concentration of single component of vaccine antigens to the preparation of aqueous phase before the emulsification with mineral oil phase. These processes yield of polyvalent vaccines containing more impure contents than those of the monovalent vaccines, leading to adverse effects on animal. Therefore, one of the challenges of the preparation of polyvalent vaccine is how to balance the reduction of antigen volume with the maintenance of the efficacy as those of the monovalent vaccine. The addition of adjuvants containing danger signals molecules to the vaccine is one effective method to overcome these drawbacks. The efficacy of the antigen-saving tetravalent with adjuvants is not influenced by the reduction of antigen contents. This indicated the adjuvants can benefit antigen-saving during manufacture process. The body weight and clinical signs showed no remarkable difference (data not shown) between the chickens in the adjuvanted and non-adjuvanted groups. This indicated no adverse effect of the adjuvant on their growth. Results also indicated that the CVCVA5 adjuvant is capable of increasing the efficacy of the vaccine with four antigens. The previous studies (Gough et al., 1977; Li et al., 2012; Yu et al., 2015) also showed the adjuvants can improve the efficacy of multivalent vaccines.

The CVCVA5 adjuvant is also significantly improved H5 and H9 antibody titers, which indicated that chickens which received the H5–H9 vaccines after the administration of ND-IB-CVCVA5 with 5 days intervals. Both of IFNγ and IL-4 in the chickens’ serum maintained high levels at 5 and 14 days PV of ND-IB-CVCVA5. The cytokine IFNγ and IL-4, elicited by the CVCVA5, may synergistically increase the immune response to ND-IB vaccine with it and the following H5–H9 vaccine without it. Other researchers (Piedrafita et al., 2013) reported the similar efficacy of adjuvant benefit to following vaccination.

In summary, this study showed the immunopotentiator CVCVA5 increased the serum and mucosal antibody levels, cytokine levels of IFNγ and IL-4 of the chickens which received the univalent vaccine, and also improved serum antibody titers in bivalent and tetravalent vaccines immunized chickens, respectively. Specifically, the vaccine-sparing and antigen-saving immunization could substantially benefit the poultry farmers, as well as the vaccine manufacturers.

## Author Contributions

Conceived and designed the experiments: YT, PW, JL, XL, and JH. Performed the experiments: YT, PW, JL, LF, XZ, and MM. Analyzed the data: YT, PW, JL, LF, XL, JH, and HW. Contributed reagents/materials/analysis tools: YT, PW, JL, XL, JH, and HW. Wrote and editing the paper: YT, PW, HW, and XL.

## Conflict of Interest Statement

The authors declare that the research was conducted in the absence of any commercial or financial relationships that could be construed as a potential conflict of interest.
